# Vertical program of screenings and check-ups in the Russian Federation: design, implementation and lessons learnt

**DOI:** 10.1186/s13690-022-00878-3

**Published:** 2022-04-15

**Authors:** Igor Sheiman, Sergey Shishkin, Svetlana Sazhina

**Affiliations:** 1 The National Research University Higher School of Economics, Faculty of Social Sciences, 20, Myasnitskaya St, Moscow, 101000 Russian Federation; 2 The National Research University Higher School of Economics, Centre for Health Policy, 4 Slavyanskaya Ploshchad, Building 2, Moscow, 109074 Russia

**Keywords:** Vertical health program, Screenings, Public health, Russian Federation

## Abstract

**Background:**

The Russian Federation has introduced a vertical large-scale program of ‘dispensarization’ (Program) that includes health check-ups and screenings for the entire adult population. It is expected to improve the results of medical interventions and ensure health gains at a relatively low cost. The major research question: Does the design and implementation of the program meet the expectations?

**Methods:**

We analyze regulatory acts and the literature on the design and the outcomes of the Program. Physicians’ surveys and interviews are conducted to understand the capacity of primary care providers to meet the requirements of the Program, as well as the link between the early identification of new illnesses and their follow-up management, administration of the program, the barriers to its successful implementation.

**Results:**

There is a substantial progress in the coverage of the population and increase in the number of identified illnesses. Some specific instruments of the Program implementation work well, others require more careful design and additional integrative and managerial activities. The capacity of primary care providers does not allow to provide high quality preventive services, as well as to ensure a continuum of preventive and curative work. The pattern of the Program administration facilitates its nation-wide implementation according to the unified rules, but makes it more difficult to account for the local conditions and limits the autonomy of professionals to choose specific population risk groups and a list of services. The interaction of providers in preventive activities is inadequate.

**Conclusion:**

The expectations of the Program are too high due to the inconsistencies in its design and implementation. The major lesson learnt is that the program like this should meet the capacity of primary care and be designed as a complex of interrelated activities to identify illnesses and provide their follow-up management.

**Supplementary Information:**

The online version contains supplementary material available at 10.1186/s13690-022-00878-3.

## Background

The majority of European countries have implemented comprehensive approaches to public health. Population-based check-ups and screenings have become an important instrument of the early identification of illnesses and their follow-up management. These activities are usually viewed as the way to improve the chances of survival for people living with cancer and other illnesses by ensuring that health services can focus on diagnosing and treating the disease earlier [1]. The population coverage by screenings is high and growing in many OECD countries [2].

The impact of these activities on health outcomes is not as easy as it might seem. It depends on the selection of preventive services, as well as on the implementation practices in the specific national context. There is a substantial body of literature on the justification of screening programs. Wilson and Jungner [3] set out ten principles of the choice of screenings, which remain the cornerstone of the literature on this issue, particularly the principle that the ratio of cost/utility should not be lower for screenings than for the curative activities. Numerous studies evaluate the expected impact of these programs on mortality and other health indicators, as well as the expected cost effectiveness of the alternative programs [4–6]. There is a growing consensus on the principle of selecting the program: ‘Screening may bring benefits but also harm; just because it can be done does not mean that it should be done’ [7].

Another body of literature addresses the design and organization of screening programs. WHO Regional Office for Europe [8] suggests core steps of screening pathway from identifying target populations to monitoring and evaluation. The major research areas include the ways individuals in the target population are identified, forms of their involvement, the appropriate management of screen positive and negative results, actors of service provision and their interaction [9].

Practically all these studies address the developments in Western countries. Much less attention is paid to the post-Soviet countries. Some of them have deeply rooted traditions of the population-based preventive campaigns. Modern Russia inherited the Semashko model, which had declared the priority of preventive activities. But their implementation after the dissolution of the USSR has been limited by chronic underfunding of health system. Public funding has not exceeded 3.5% GDP over the last three decades [10]. In the early 2010s, the priority of prevention campaigns has been re-vitalized in the form of a nation-wide vertical program of ‘dispensarization’ (further Program), that is a set of preventive activities, including health check-ups and screenings. This is a term from the original Semashko model, practically unknown in the international literature.

The expectations of the Program are very high in Russia. The official attitude is that the early detection of ill health will allow to improve the results of medical interventions and ensure health gains at a relatively low cost. The Federal Ministry of Health (MoH) estimates the contribution of the prevention campaign to ‘keeping people healthy’ at the level of 60% [11]. Also, the Program is viewed as a tool that will decrease the need for curative work of primary care providers. The MoH predictes a fall of curative visits share from 60 to 40% of all primary care physicians visits with a corresponding increase in the share of preventive visits [12]. Such optimistic expectations explain a substantial involvement of the government in the implementation of the Program.

The Program design provides for the specific instruments of its implementation – highly centralized administration, a universal set of services to identify illnesses among centrally determined target groups, methods of planning, reporting, monitoring, etc. Some of these instruments, for example, provision of preventive services in multi-specialty primary care settings, a large-scale promotion campaign, the establishment of special units responsible for this work, are not common internationally.

The recently implemented policies to strengthen health prevention in Russia have prompted a number of questions: Does the design and implementation of the Program meet the capacity of the current health system? Do specific Russian instruments really work? Does a highly centralized pattern of the Program administration facilitate or complicate its successful implementation? Which lessons can be learnt from this Program for other the countries? Addressing these questions may be of interest to health policy makers in other countries seeking the ways to improve public health. We explore these questions by reviewing the design of the Program, studying its implementation practices and discussing the results. The selection of specific check-ups and screenings is not discussed in the paper, since it is country-specific and is beyond our research questions.

The motivation to produce this paper is to encourage a more careful study of population-based prevention programs, particularly in countries with limited financial resources for health care. Their policy makers often seek ways to solve health care problems through the early identification of new illnesses. The major message is that this activity does not automatically yield health gain. The population-based programs of check-ups and screenings should meet the capacity of primary health care. They should be designed as a complex of interrelated activities to identify illnesses and provide a follow-up management of chronic cases. Physicians should have a discretion regarding the choice of prevention patterns, including the coverage of specific population risk groups and a list of check-ups and screenings. A close interaction of providers in the course of new diseases identification is also needed.

Thus, the aim of the paper is to analyze the design, implementation and outcomes of the Program in the context of domestic and international developments in public health.

## Methods

**Fig. 1:**
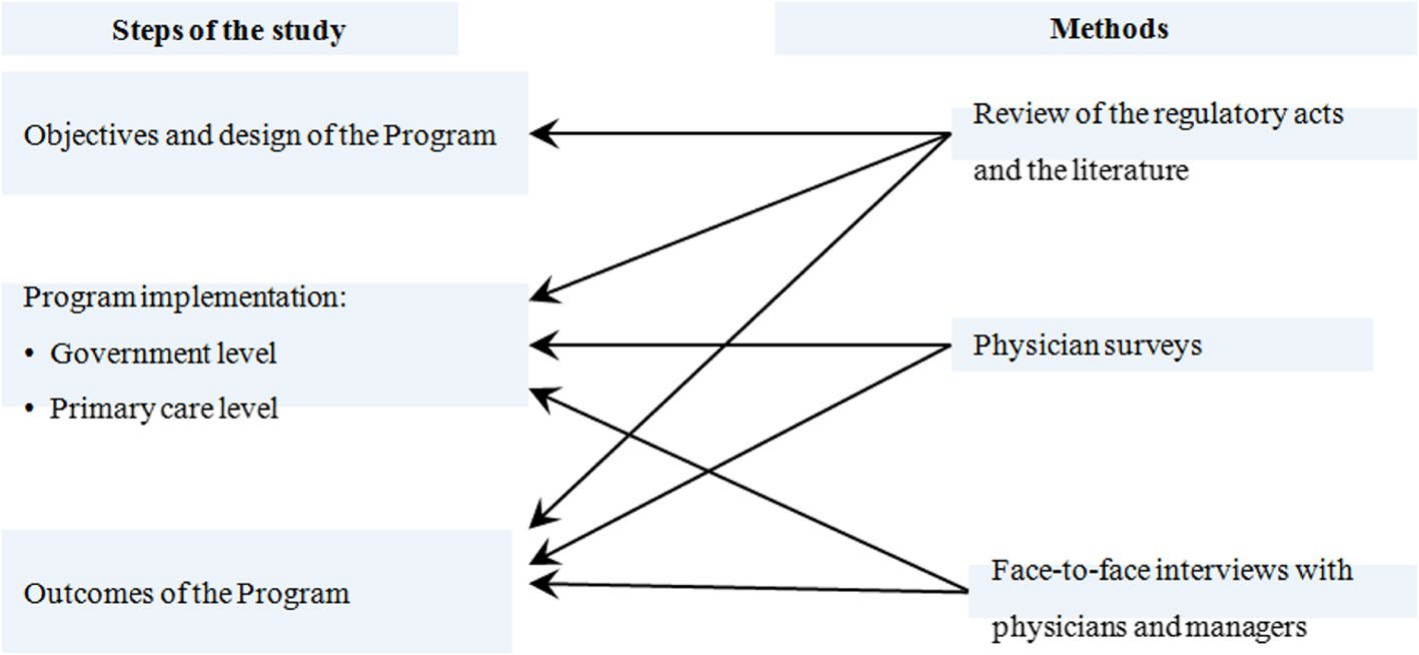
Study design and methods

We analyze design, implementation and outcomes of the Program using as research tools the review of regulatory acts and the literature, statistical analysis, physician surveys, face-to-face interviews with physicians and health managers. A framework of the analysis is presented in Fig. 1.

### Study design

We follow a three-step methodological framework. The first step involves the analysis of objectives and design of the Program. The regulation is explored to determine the preventive activities requirements and their matching standard pathways recommended by WHO. We explore the patterns of check-ups and screenings provision, planning preventive activities, reporting outcomes, monitoring, payment methods. The limitations of the design are highlighted.

The second step addresses the issues of the Program implementation. It consists of two stages. The first is to highlight the administration of the program at the governmental level. The second is to explore the implementation of the Program at the level of primary health care (PHC) settings. The focus is on the following characteristics of their performance: a) the adequacy of primary care capacity to implement the Program; b) the interactions between providers in terms of the flow of information on the early identification of new illnesses; c) the prevalence of the follow-up management of these illnesses, d) the modes how providers of preventive services choose target groups for screening, plan their activity, report the outcomes. These parameters of implementation are studied with the use of physician surveys and face-to-face interviews.

The third step involves the analysis of outcomes of the Program as they are defined in the regulatory acts. The official estimates of the population coverage and the identification of new illnesses are compared with the estimates of physicians obtained through the survey. The collected evidence allows us to come up with concluding points about the potential of the Program to meet the public expectations.

### Data sources and instruments

To explore the objectives and the design of the Program, we analyze the regulatory acts of the government and the domestic literature on the subject, including the reports in limited circulation, unpublished documents, memorandums, and presentations from our personal collections. The literature on the similar programs in other countries was searched in the MEDLINE data base using the query “screening”, “health check-up”, “public health programs”. All findings were checked manually and around 30% were relevant.

The practices of the Program implementation were explored using a physician survey. It was conducted online in April–May 2019 through the mobile ap “Handbook of Physician” (available in Google Play and AppStore) with 540 thousand of registered users. We randomly selected 1100 physicians and feldshers (paramedics) who were directly involved in provision of preventive services in primary care settings under the Program, including district therapists (63.2% of respondents), outpatient specialists (18.7%), general practitioners (9.9%), paramedical personnel (8%). They represent most of regions (81 out of 85 regions of the country) and the structure of population residence (658 physicians from regional centers, 172 – other urban areas, 273 – from rural areas). A questionnaire with 25 questions on the above mentioned dimensions of implementation practices was sent to the selected group of respondents (appendix 1). Only part of the survey results is presented in the paper due to its limited space.

To understand the degree of professional autonomy on the choice of target population groups and specific preventive services, we conducted a small-scale survey of 103 primary care physicians and outpatient specialists in three big multi-specialty urban polyclinics in Moscow city and Moscow oblast (the region around the capital) in January 2020 when Covid-19 pandemic had not affected the work of primary care yet. We explored the ways preventive activities were administered in polyclinics with 13 questions (appendix 2). Ten physicians on the list were approached directly for face-to-face interviews.

We also conducted face-to-face interviews with two high-level managers of private medical companies that were involved in the realization of the Program. The main interview topics were: What is the estimate of the actual coverage by the program?” “Can primary care physicians select population groups and their own ways to conduct health check-ups|?” “Does centralized administration of the Program facilitate or complicate its successful implementation?” “What is needed to ensure a follow-up management of patients with the early identified diseases?” The interview data were compared with the results of public opinion surveys conducted by polling companies.

The analysis of the Program implementation and outcomes is based on the statistical data and reports of the federal MoH and the estimates of physicians.

## Results

### Objectives and design of the program

#### Objectives

The regulatory document of the Federal Ministry of Health [13] defines the dispensarization as ‘a complex of activities that includes health check-ups and additional methods of investigations conducted with the aim of evaluating health status … of targeted groups of population’. The Program pathway is similar to the one recommended by WHO for screening programs: identification of the population eligible for screening, invitation and information, testing, referrals to specialists, diagnosis, follow-up management of identified cases, reporting the outcomes [7]. Most of these steps are in place in the Program, except for the follow-up management. The latter is mentioned in the regulatory act as a so called ‘dispensary surveillance’. This term of the original Semashko model means that every identified case of a serious disease is subject to a certain set of clinical protocols (which is close to the modern programs of chronic disease management). However, the follow-up management of the case does not constitute a component of the Program. One of its objective is formulated as ‘establishing the group of dispensary surveillance of patients with chronic diseases and high risks of circulatory system diseases”, that is determining the need for such a surveillance rather than its actual provision. The regulation also sets the requirement of distributing participants into three health groups according to the severity of identified illnesses.

Thus, the Program is viewed as the way to ensure the early identification of illnesses and prevention of their complication. The dispensarization process for an individual is finalized with a documentation of medical examination results, assigning to a health group and some awareness of health problems.

#### Patterns of health check-up and screening provision

A set of preventive activities is based on the evidence of their outcomes collected by research institutions. A universal set of these activities are determined for the entire country. The process of dispensarization for an individual patient consists of two stages. The first stage is aimed to detect risk factors and deviations in a patient’s health. The second stage is to confirm or reject the first stage findings. Patients are referred to specialists for consultation and/or additional tests. These services are provided to the target age and sex groups of population. People older than 40 years in all regions of the country are supposed to go through a required set of check-ups and screenings once a year. The group of 18–39 years old – once in three years. Most of children go through only the stage of check-ups.

The design of the Program is based on the assumption that most preventive activities are provided in the same outpatient setting – a multi-specialty polyclinic with 10–15 categories of outpatient specialists in big urban areas, 3–5 categories in small cities and the number of people served ranging from 30 to 300 thousand people. The major providers of the first stage activities for adults are district therapists and GPs, for children – district pediatricians (together they are further referred to as district physicians – DPs). In big polyclinics, they are reinforced by the staff of a preventive unit – physicians and nurses responsible only for preventive activities. Such units exist in practically all urban polyclinics [14]. Specialists deal with the second stage of the Program. Rural and small town areas are served mostly by solo practices (physician ambulatories). They take on preventive activities of the first stage and refer patients to urban polyclinics for the second stage activities. Feldshers do this work in the smallest rural areas.

#### Planning and reporting the outcomes

The administration Program is highly centralized and implemented by the federal Ministry of Health. The requirements of planning and reporting are based on the federal regulation. The major indicator is the coverage of the eligible citizens. It is planned vertically – for the entire country, regions, communities, medical organizations, district units served by DPs. Also, the Federal Program of state guarantees of free medical care – the major health planning and funding document – sets the targets for the number of physician visits with the “preventive aim” and their unit cost. Using these targets, regions plan their own volumes of preventive care and their funding with the following distribution of these volumes across communities and polyclinics.

In addition to the population coverage, the prevalence of detected cases of illnesses is reported through a vertical chain of governmental agencies and medical settings. This reporting focuses on the cases of circulatory system diseases and cancer. In the latter case, it includes the indicators of cancer identification at first and second stages. Some regions of the country plan the number of new cases identified in the course of the Program implementation and even set targets for this indicator.

#### Monitoring the program

It follows the lines of planning and reporting. The major indicators of success are a high and growing population coverage, a high share of the early identified cases, and a high share of the identified cases that is subject to dispensary surveillance has been added. However, monitoring the actual follow-up curative activities is not required by the regulation, let alone monitoring the outcomes of dispensary surveillance. The actual health gains of the Program are beyond the scope of monitoring.

In addition to the aggregate information, detailed data is collected: the number of specific services, detected illnesses, prevalence of risk factors, the number of patients who need a dispensary surveillance, health groups and their structure. Theoretically, this data allows to analyze the outcomes of preventive activities across stages of the Program, specific screenings, groups of population, geographic areas. But the regulation does not require such a detailed monitoring. Moreover, this information is not open for the public.

#### Payment method

Preventive services under the Program are reimbursed by regional mandatory health insurance (MHI) funds. The rates of payment are set for a so called ‘finished case of dispensarization’, that is a fixed package of services determined by the regulation for the first stage of the Program. A ‘luft’ of 15% of the number of services is allowed, while all screenings are obligatory. A bundled payment for the first stage is supplemented with a fee-for-service reimbursement of additional consultations and tests at the second stage. The control of the actual number of ‘finished cases’ is conducted by health insurers – mostly private entities that are involved in MHI. In addition to this amount, polyclinics receive a small bonus (around 13–15 USD) for each identified new oncological case [15]. The revenue of polyclinics under the Program is linked to the planned volume of preventive activities. If the actual volume is lower, then the amount of funding is lower than planned.

The idea behind this pattern of payment is to motivate providers to supply preventive services. The opposite side of the coin is that this instrument limits the professional autonomy of physicians on the choice of preventive services for an individual. They have to provide the entire bundle of services to be reimbursed, irrespective of the actual need of a patient. Inversely, a necessary test that is not included in the list of the ‘finished case’ will not be paid. Also, the regulation does not provide for additional payment for managing identified chronic cases. Dispensary surveillance is not incentivized by payment methods. This is another limitation of the Program design.

### Implementation of the program

#### The role of the government

The role of a highly centralized administration of the Program is controversial. On the one hand, the federal government has initiated it, provided regions with additional funding, involved providers in preventive activities, made the Program implementation a priority of health policy. The opportunities and benefits of preventive activities are widely presented in the state media and official websites with the focus on the information how and where to pass medical examination. Private employers are legally required to promote the involvement of their employees and to offer them a day-off once a year to undergo the dispensarization. In some regions, temporal offices for check-ups have been established in popular trade and recreation zones, as well as in big educational institutions and industrial centers. In addition to the support activities, administrative pressure is used wherever possible. Public servants, teachers, medical workers, students and some other groups of population are strongly recommended to participate in the campaign. There are voices to introduce financial sanctions for those who ignore the Program. All these strategies contribute to the population coverage.

On the other hand, a highly centralized administration has a number of drawbacks. Uniform target population groups and a set of preventive activities limit the flexibility of regions in responding to local needs and special conditions – variation in the disease incidence, the capacity of PHC, the most vulnerable population groups. Centrally established indicators of the population coverage, volumes of preventive care and the number of identified illnesses make regional health authorities and PHC providers look for the ways to reach the targets irrespective of the local capacity to treat new cases. Monitoring the Program outcomes follows the logic of the centralized administration and politically loaded campaigns. The federal MoH makes an emphasis on easily attainable indicators and targets so that to show the progress.

Health managers in their face-to-face interviews indicate that the centralized administration of the Program complicates the actual preventive work. Plans of the population coverage don’t take into account the age structure of eligible groups. If the share of elderly people is high, then the planned targets can’t be reached, because elderly people usually know enough about their illnesses and prefer to see a doctor for curative purpose rather than to have check-ups. If the targets can’t be reached, physicians have to look for the ways how to distort reporting about the coverage of the program. Also, the requirements of the federal MOH often change, which in turn requires changes in electronic forms for medical examinations, procedures of reporting and billing, etc. The citations of interviews:

*“Rigid plans for medical examinations turn dispensarization into a competition of numbers and indicators that are not related to the reality”.*

*“Permanently changing federal rules of dispensarization divert physicians from the actual preventive work and overburden them with routine reporting work”.*

*“Tough federal requirements and plans force providers to distort reports of the coverage and identification of illnesses”.*

#### The performance of primary care providers

##### A low capacity of primary care

The Program has been implemented in the health system where DPs are overburdened with a curative work. The survey of 1100 physicians (appendix 1) indicates that 63.9% of district therapists serve more adults than a normative workload of physician established by the MoH – 1700 adults per district therapist. 21.7% of respondents report that they exceed this target nearly by two times.

Our statistical estimate indicates that the average size of the catchment area per district therapist in the country is 2900 adults, while in some regions – 3–4 thousand [16]. The estimate of the deficit of district therapists to meet the normative workload is 33%, district pediatricians – 19% [17]. Their task profile has increased substantially due to the introduction of the Program. In many medical settings, they have to substitute a usual curative work for check-ups and screenings which are mandatory and closely controlled by administrators of all levels.

##### The interaction of providers

The implementation of the Program in urban areas is based on the model of a big multi-specialty polyclinic. The major strength of this model is its capacity to provide comprehensive preventive and curative care. Patients can undergo check-ups and screenings, see DPs and specialists ‘under one roof”. Also, a polyclinic model is expected to demonstrate the additional leverage to implement integrated care pathways. But to make this happen, specific integrative activities are needed. Our previous studies indicate that they are lacking in the curative work [18]. They are equally lacking in the area of preventive services.

**Fig. 2:**
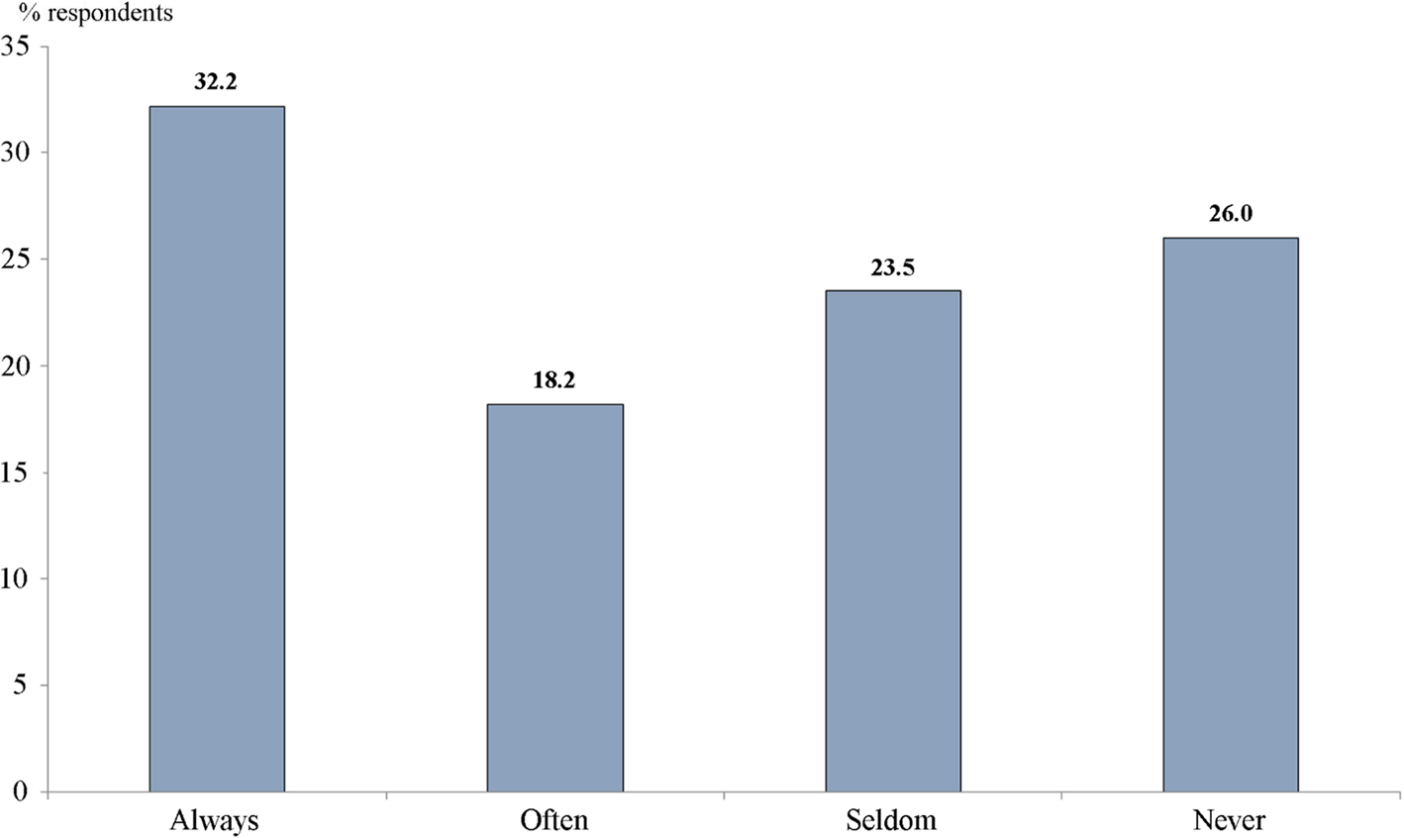
Distribution of responses to the question ‘Do you receive information about results of medical examinations of your patients under the program of dispensarization when they are conducted by preventive units?’ % of all respondents

The major problem is a poor flow of information about identified illnesses between professionals involved in the implementation of the Program. According to the survey, nearly two thirds of respondents (63.8%) report that health check-ups are conducted by DPs, 13% – by physicians of preventive units, 22.4% – jointly. Only half of DPs always (32.2%) or often (18.2%) receive information about check-up results when they are conducted by preventive units. The rest seldom or never receives it (Fig. 2). This is a sign of a blurring responsibility for check-ups between DPs and preventive units, as well as a poor interaction between them.

As indicated above, the Program provides for the distribution of the eligible population across health groups. This is an important information for district physicians who are responsible for their patient list. However, the survey indicates that more than half of DPs (53%) are unaware of the distribution of their patients across health groups. Check-ups and screenings are conducted, patients with health problems are identified, but many physicians responsible for their follow-up management don’t know about results of preventive activities.

Another area of providers’ interaction is between DPs and specialists. Every third DP (34.2%) seldom receives information about the results of the second stage dispensarization, every fourth (24.3%) doesn’t receive it at all. Thus, more than a half of DPs don’t report coordination with specialists involved in the Program.

##### The patterns of the follow-up management of identified illnesses

**Fig. 3:**
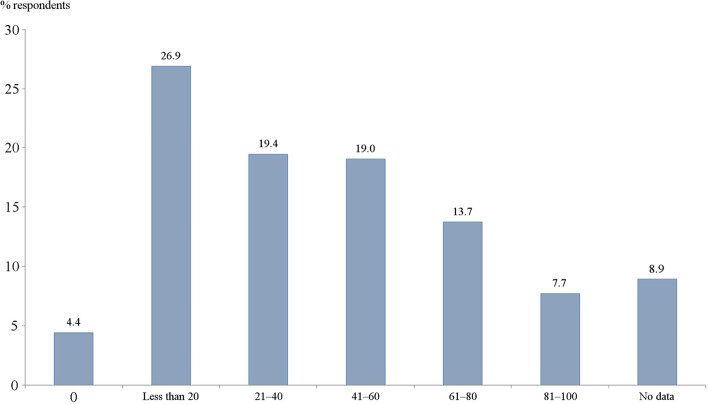
Distribution of responses to the question ‘What is an approximate share of patients (who are assigned to a DP) is managed according to the requirements of the pattern of dispensary surveillance issued by the Ministry of Health?’ % of all respondents

Only 7.7% of respondents indicate that a set of actual curative activities meets the requirements of a pattern of dispensary surveillance issued by the Federal MoH. The majority reports that these requirements are met only for some patients or are not met at all (Fig. 3).

The usual practice to evaluate the outcomes of chronic disease management is to look at the number of disability days, emergency care visits, hospital admissions, disease-specific mortality rates [19]. In this research, we tried to explore a trend of these indicators according to the estimates of the respondents. A decrease in the number of disability days of chronic patients is reported by only 14% of physicians. More than a half of respondents are unaware of the number of emergency care visits and hospital admissions of their chronic patients.

These findings are similar to a clinical expertize conducted by the private health insurer ‘Rosgosstrah-Medicina’. The analysis of medical records of 7043 patients after their hospital admissions with a stroke or a myocardial infarction indicates that nearly half of these patients have not seen a doctor during the year prior to admission [20]. The nation-wide survey of physicians in late 2018 demonstrate that 72% of respondents agree with the point that the Program has not contributed to the management of the identified new diseases [21].

The revealed interaction of preventive and curative activities does not meet internationally recognized assumption that a screening program pathway should include a stage of the follow-up management of the detected illnesses: ‘ … there is no point in offering a screening program if there are insufficient facilities or health personnel to provide treatment for those who need it’. For example, a mass screening program for thyroid cancer in South Korea in 1999 led to the number of cases being detected increasing 15-fold and yet no reduction in mortality from thyroid cancer [7]. In Russia, the Program is focused on the identification of new illnesses. It is supposed that a follow-up treatment will be organized in the course of usual performance of health providers. But as indicated above, the principle of continuum of preventive and curative activities is observed only for a part of identified cases.

##### The patterns of the program administration in primary care settings

A centralized Program administration sets a chain of vertically determined rules. Physicians involved in the implementation of the Program work according to the rules determined by their polyclinics’ administrators, who in turn follow the commands of the federal and regional health authorities. The following evidence of physicians’ limited professional autonomy is collected in the survey of 103 physicians of multi-specialty polyclinics in Moscow city and Moscow region:

- 66% of physicians report that they have individual plans of the number of preventive and curative visits, developed by polyclinics administrators. Only 34% plan their activity themselves;
- 59% indicate that the failure to implement individual plans on the number of visits may cause reduction in their remuneration;
- only 25% of physicians select patients themselves for check-ups and screenings after assessing their risk factors, that is invite those with the highest risks. The majority relies on the centrally determined target population groups.

Administrators of multi-specialty polyclinics plan the number of visits and the Program coverage in the catchment areas, control the activity of physicians. The professional autonomy of physicians to select their own patterns of preventive work is limited. The face-to-face interviews with physicians confirmed this conclusion.

## The outcomes of the program

As stated above, the major outcome indicator in the official reporting is the coverage of the eligible population. The Federal Fund of MHI reports that in 2018 92% of eligible population has actually undergone dispensarization, in 2019–110% [22], while the latter estimate probably accounts for those who passed medical examinations more than once. However, physicians are less optimistic. According to the survey of physicians, more than half (51.4%) report that this share is less than 60%, while 17.4% think that the coverage is less than 20%.

We also asked a question ‘Why do you think there is a common opinion in the society that the number of people that have actually undergone dispensarization is lower than official estimates?’ Nearly two thirds of physicians (62.6%) report that the major reason for this opinion is that people are reluctant to undergo dispensarization, therefore physicians have to report an upward distortion of the coverage.

The estimates of the number of identified illnesses are based on the reports of the federal MoH for individual years, the reliability of which cannot be verified due to the unavailability of the original accounting information. Particularly impressive are reports of an increase in the number of diagnosed oncological diseases - from 21.3 thousand in 2013 to 55 thousand in 2019, or by 2.6 times. The frequency of newly diagnosed breast cancer in the period 2013–2018 increased from 39.2 per 100 thousand in 2013 to 73.3 per 100 thousand in 2018 or by 1.9 times. For other cancer localizations, the data for individual years are incomparable. In 2018, the MoH gave estimates of the proportion of cancer detected at stages 1–2: cervical cancer - 67.9%, breast cancer - 68.7%, colorectal cancer - 57.1% [17]. Comparable data for other years are not available.

According to the same source, the number of diagnosed cardiovascular diseases increased from 1.6 million in 2014 to 8.5 million in 2018, or 5.3 times. Particularly impressive is the dynamics of the diabetes mellitus detection: in 2015, more than 300 thousand cases were detected, in 2016 - already 531 thousand cases, or 1.8 times more. Approximately the same picture is emerging for respiratory diseases: there was an increase in the number of detected cases from 500 thousand to 710 thousand, or by 40% in one year [17].

Health groups characterize the distribution of the population according to the prevalence of pathologies of varying complexity. The share of the third group with the lowest health status increased from 44% in 2013 to 54% in 2018 [23].

Thus, the available official data indicate a significant increase in the coverage of medical examinations, a high rate of disease identification, an increase in the proportion of the population with serious diseases. As indicated above, the official estimates of the coverage are disputed by physicians.

## Discussion

The Program of dispensarization in Russia has raised the priority of the population-based preventive activities. The early identification of illnesses is currently viewed as one of the major areas of health policy. Primary care providers are increasingly focused on delivering check-ups and screenings to identify new cases. This work is supported by the government. There is a substantial progress in the coverage of the population and the number of identified illnesses. However, the collected evidence demonstrates major issues that don’t allow to expect substantial health gains – a low capacity of primary providers, the weakness of the follow-up management of identified illnesses.

The major barriers to successful outcomes of the Program reflect the characteristics of the current health system governance in Russia. It is highly centralized. Democratic institutions are very weak. The role of regional governments in choosing the priorities of health policy is marginal. Local communities and professional organizations are rarely involved in decision-making on health issues. Patient empowerment practically does not exist. The input of civil society bodies is largely imitative. Therefore, the design of the Program has not been publicly discussed and it has not been adjusted to the low capacity of PHC, as well as to the requirements of holistic provision of preventive and curative services. In the pandemic of COVID-19, the Program has been suspended but will most likely be re-started in the unchanged form.

The study has a number of limitations. First, the section on the Program outcomes is based mostly on the official estimates that overestimate the positive input of dispensarization. The reports of the federal MoH claim that check-ups and screenings are the major contributors to the early detection of diseases [11, 12], but this seems to be a strong exaggeration. The estimates of Russian oncologists indicate that the input of the Program to the total number of newly diagnosed cases of oncological diseases is relatively low and growing only slightly: in 2013 it was 25%, and in 2018–36%. The major part of these cases can be accounted to the regular curative work [24]. The insufficiency and unreliability of the data make our survey-based estimates preliminary. Second, our research was not designed to assess: a) the impact of specific tests and screenings on the identification of new cases, b) the actual coverage, specific activities and health outcomes of the follow-up dispensary surveillance, c) the cost of specific preventive services. Third, the cost effectiveness of the entire Program and its elements is beyond the scope of the research. These are the areas of future research.

The design of two physician surveys, as it is seen in questionnaires (appendixes 1, 2), has allowed to receive interesting results on a wide range of the Program characteristics. Some of them are not presented in this paper due to its limited space. For the same reason, some qualitative assessments are not supported by figures and tables with distribution of responses.

Report bias of the nation-wide physician survey relates to the question about the common opinion that the official estimates of the Program coverage are too high. Most respondents agree with this opinion but attribute it to the reluctance of eligible population to participate in the Program. A logical link between the two is dubious. Physicians tend to distort reporting under the pressure of administrators who are keen about reaching targets. This is a major reason for the overestimate of the coverage.

There is some bias in the design of the survey of 103 primary care physicians in two regions of the country. They definitely don’t represent all physicians in the country. The only justification for this small-scale survey is that it is related to a narrow and more or less clear area of professional autonomy limitations. We wanted to receive an additional evidence without conducting a large-scale survey.

No single aspect of the research would be sufficiently robust on its own, but the combination of literature and regulatory acts analysis, physician survey, face-to-face interviews, national public opinion surveys and statistical data create a rich picture of how the Program is designed and implemented, what are the barriers to achieving positive outcomes. There are grounds to believe that the generalizability of findings is high.

## Conclusion

The Program of dispensarization has increased the coverage of the population with check-ups and screenings. The Program aims at increasing the number of identified illnesses with a low priority of the follow-up management of new cases. The empirical evidence indicates that the capacity of primary health care does not allow to provide check-ups and screenings, as well as to ensure a continuum of preventive and curative work. A highly centralized pattern of the Program administration facilitates its nation-wide introduction and implementation according to the unified rules, but makes it more difficult to account for local conditions. The balance between centralized governance and the right of regions to adjust the Program to the local conditions has not been reached yet. This is an important task for any large-scale prevention campaign. Some specific instruments work well, particularly the establishment of preventive units in primary care settings, forming health groups of the eligible population, large-scale support and promotion of preventive campaign by the government. Others require more careful design and additional integrative and managerial activities.

The major lesson learnt from this bold experiment is the understanding of insufficiency of a simplistic approach to health care problems by increasing the priority of the early identification of illnesses with the expectation that this may become a ‘magic tool’ of health improvement. The expectations of the program are too high, but there is a lack of consistency in its design and implementation. The population-based programs of check-ups and screenings should meet the capacity of the health care and be designed as a complex of interrelated activities to identify illnesses and provide their management with the focus on chronic cases.

## Supplementary Information

**Additional file 1.**

**Additional file 2.**

**Additional file 3.**

## Data Availability

The datasets used and analyzed during the current study are available from the corresponding author on reasonable request.

## Abbreviations

OECD: Organization of Economic Cooperation and Development
WHO: World Health Organization
MoH: Ministry of Health
Program: Program of dispensarization in the Russian Federation
PHC: Primary health care
GP: General practitioner
DP: District physician
MHI: Mandatory health insurance
