# Supplementary material for: Vertical program of screenings and check-ups in the Russian Federation: design, implementation and lessons learnt
Source: Arch Public Health. 2022 Apr 15;80:123. doi: 10.1186/s13690-022-00878-3 (PMC9012158; doi:10.1186/s13690-022-00878-3)
Supplement: Supplementary file 1 — Additional file 1. [file 13690_2022_878_MOESM1_ESM.docx]

**Appendix 1.** Q**uestionnaire of a physician survey on the implementation of the Program of dispensarization in Russia (1100 primary care physicians)**

1. Your region of residence? ____________

2. Your gender ____________

– male

– female

3. Your year of birth. __________

4. What position do you hold?

– District therapists

– GP

– Other (please enter it manually)

5. Your clinic is located:

– in the administrative centre of your region

– in another city of your region

– in rural area

6. Number of patients in your district (please enter it manually) _____________

7. Who conducts regular medical examination of the population attached to your polyclinic, as part of the dispensarization?

(You can choose both options)

– District therapists / GP

– Physicians of preventive units

8. Which of the following scenarios most often becomes the starting point for a citizen to undergo the dispensarization? (one answer)

– Direct self request of a citizen to a district physician for the purpose of undergoing the dispensarization

– Direct self request of a citizen to a preventive unit for the purpose of undergoing the dispensarization

– Calling a citizen by a doctor to undergo the dispensarization

– Visit of a citizen to a district physician regarding a disease

– Visit of a citizen to a specialist regarding a disease

– Other (please enter it manually)

9. What is the approximate share of eligible citizens who applied for dispensarization themselves in 2018?

Please enter it manually, % ____________

10. Do you receive information about the results of preventive medical examinations of your patients when they are carried out by preventive units? (the question to district physicians)

– Always

– Often

– Rarely

– Never

11. What is the share of eligible population that actually underwent medical examination in 2018?

Please enter it manually, % ___________

12. Why do you think it is widely believed that the number of citizens who have actually undergone dispensarization is less than that presented in official estimates?

(You can choose several answers)

– Citizens do not trust statistics

– Citizens do not remember when they underwent medical examination

– Citizens do not want to undergo a medical examination, and doctors are forced to make “fake registrations” in order to improve reporting rates

– Other (please enter it manually)

13. What approach to coverage of the population for dispensarization do you consider correct?

– Total

– Selective (having higher risk for certain diseases)

14. Are there any additional payments for the implementation of dispensarization plan for the attached population?

-Yes

– No

– Other (please enter it manually)

15. Do you know the distribution of the population attached to you as district physician, by health groups based on the results of the dispensarization?

– Yes

– No

16. Do you receive plans of patient numbers under dispensary surveillance?

– Yes

– No

17. What is the approximate proportion of the population attached to you as a district physician, among those who underwent dispensarization in 2018, was taken for dispensary surveillance?

Please enter it manually, % __________

18. How are patients under dispensary surveillance distributed between you and other specialists (endocrinologist, oncologist, etc.)? What is the approximate proportion of all these patients under your supervision?

Please enter it manually, % ____________

19. What is an approximate share of patients (who are assigned to a DP) managed according to the requirements of the pattern of dispensary surveillance issued by the Ministry of Health (December 21, 2012 No. 1344n)?

Please enter it manually, % _____________

20. Do you receive information about the results of dispensary surveillance of patients attached to you as to a district physician, in cases when they are carried out by specialists?

– Always

– Often

– Rarely

– Never

21. Are there any additional payments for conducting dispensary surveillance?

– Yes

– No

– Other (please enter it manually)

22. Do you receive information about the calls for emergency care by patients attached to you as a district physician?

– Always

– Often

– Rarely

– Never

23. Were there any changes in the following performance patterns of dispensary surveillance in your district in 2018?

(You can choose several answers)

– Reduced number of days of temporary disability of patients under dispensary surveillance

– Reduced number of hospital admissions of patients under dispensary surveillance due to exacerbations and complications of diseases

– Reduced mortality of the patients under dispensary surveillance

– Reduced frequency of exacerbations of chronic diseases among patients under dispensary surveillance

– Reduced number of emergency calls among the patients under dispensary surveillance

– None of the above

– Evaluation of dispensary surveillance according to the listed criteria is not carried out at all

25. What do you think needs to be done to improve outcomes of dispensary surveillance?

(You can choose several answers)

– Reduce the workload of district service doctor to the established norm by disaggregating physician districts

– Introduce the evaluation of dispensary surveillance with indicators of reducing the population's need for certain types of medical care (emergency, inpatient care) and social care, rather than with process indicators (number of examinations, servicers, etc.)

– Strengthen the economic motivation of district physicians to increase the effectiveness of dispensary surveillance of patients

– Other (please enter it manually)
