# Supplementary material for: Vertical program of screenings and check-ups in the Russian Federation: design, implementation and lessons learnt
Source: Arch Public Health. 2022 Apr 15;80:123. doi: 10.1186/s13690-022-00878-3 (PMC9012158; doi:10.1186/s13690-022-00878-3)
Supplement: Supplementary file 2 — Additional file 2. [file 13690_2022_878_MOESM2_ESM.docx]

**Appendix 2.** Q**uestionnaire of a small scale physician survey on the administration of the dispensarization Program in primary health care settings (mostly multi-specialty polyclinics) in Russia (103 primary care physicians)**

1. Do you receive individual plans of patient visits number issued by the administration of your polyclinic?

Yes

No

Usually

Not sure

2. Does the polyclinic administration monitor the execution of these plans?

Yes

No

Usually

Not sure

3. Does the execution of these plans affect your salary size?

Yes

No

Not sure

4. Is administrative penalty used when physicians of your polyclinic regularly violate an established norm of patient visit duration?

Yes

No

Not sure

5. Does the polyclinic administration involve you in making decisions to improve the polyclinic performance?

Yes

No

Sometimes

Not sure

6. Selection of the target population for medical examination is carried out in your polyclinic:

6.1 by a doctor himself/herself with the account of risk factors

Yes

No

Not sure

6.2 in accordance with the coverage plans issued by the polyclinic administration

Yes

No

Not sure

6.3 in accordance with the requirements of the orders of the Ministry of Health

Yes

No

Not sure

6.4 other factors (please enter it manually)

Yes

No

Not sure

7. Selection of the target population for dispensary surveillance is carried out in your polyclinic:

7.1 by a doctor himself/herself with the account of characteristics of patient's condition and the course of the disease

Yes

No

Not sure

7.2 in accordance with the coverage plans issued by the polyclinic administration

Yes

No

Not sure

7.3. in accordance with the requirements of the orders of the Ministry of Health

Yes

No

Not sure

7.4 other factors (please enter it manually

Yes

No

Not sure

8. The procedure of patients referral for magnetic resonance tomography, computer tomography, echocardiogram, endoscopic examinations and similar instrumental examinations includes:

8.1 Mandatory alignment with head of the polyclinic department

Yes

No

Not sure

8.2 Mandatory alignment with medical director of polyclinic

Yes

No

Not sure

8.3 Mandatory alignment with the medical commission of polyclinic

Yes

No

Not sure

8.4 Other alignment (please enter it manually)

Yes

No

Not sure

8.5 Alignment is not required

Yes

No

Not sure

9. The procedure of referring patients to hospital admission includes:

9.1 Mandatory alignment with head of the polyclinic department

Yes

No

Not sure

9.2 Mandatory alignment with medical director

Yes

No

Not sure

9.3 Mandatory alignment with the medical commission of polyclinic

Yes

No

Not sure

9.4 Other alignment (please enter it manually)

Yes

No

Not sure

9.5 Alignment is not required

Yes

No

Not sure

10. The procedure of prescribing free medicines includes:

10.1 Mandatory alignment with head of the department

Yes

No

Not sure

10.2 Mandatory alignment with medical director

Yes

No

Not sure

10.3 Other Alignment (please enter it manually)

Yes

No

Not sure

10.4 Alignment is not required

Yes

No

Not sure

11. How often are your decisions on managing patients based on:

11.1 Your assessment of the medical situation, individual characteristics of a patient and the course of the disease (one answer)

Rarely

Sometimes

Usually

Not sure

11.2 Requirements of clinical guidelines that are focused on the average patient

Rarely

Sometimes

Usually

Not sure

11.3 Standards of medical care

Rarely

Sometimes

Usually

Not sure

11.4 Other (please enter it manually)

Rarely

Sometimes

Usually

Not sure

12. Do you experience excessive regulation of your professional decisions in the following areas:

12.1 Determination of the visit duration

Rarely

Sometimes

Usually

Not sure

12.2 Choice of medical examination

Rarely

Sometimes

Usually

Not sure

12.3 Choice of physician for referral

Rarely

Sometimes

Usually

Not sure

12.4 Choice of a patient management strategy

Rarely

Sometimes

Usually

Not sure

13. Do you see the correlation between the level of your salary and the income of the polyclinic?

Yes

No

Not sure
