# Supplementary material for: Vertical program of screenings and check-ups in the Russian Federation: design, implementation and lessons learnt
Source: Arch Public Health. 2022 Apr 15;80:123. doi: 10.1186/s13690-022-00878-3 (PMC9012158; doi:10.1186/s13690-022-00878-3)
Supplement: Supplementary file 3 — Additional file 3. [file 13690_2022_878_MOESM3_ESM.docx]

**Point-by-point response to reviewer 2**

Dear Dr. Oliver Bruyere

Thank you for the valuable comments, particularly comments on the weakness of the structure. We agree with them and restructured the paper according to a new methodological framework. We also revised some sections of the paper, as recommended. See below.

The comments of the reviewer 1 are positive. We have not found the comments to respond.

Best regards

Igor Sheiman

Sergey Shishkin

Svetlana Sazhina

| **Comments** | **Responses** |
| --- | --- |
| **Methods section** | |
| The Methods section could be better structured (e.g. included subheadings), visual support could be added here. | Agree that the methods section is poorly structured, therefore: a) gave a methodological framework of the analysis, b) included subheadings (study design and data sources and tools), c) added visual support of the study design and methods |
| Overall summary of the methodology should be provided in the first paragraph. | We start with the paragraph: “We analyze design, implementation and preliminary outcomes of the Program using as research tools the review of regulatory acts and the literature, statistical analysis, physician surveys, face-to-face interviews with physicians and health managers. A framework of the analysis is presented in fig 1” (p.5). |
| The authors also could include the Survey instrument and Themes | We presented questions of two survey in appendixes 1 and 2 as supplementary materials. The themes and specific questions of face-to-face interviews are given in methods sections. |
| One other limitation of this section is a lack of theoretical framework behind the approach to the implementation research. There is substantial literature related to the implementation research, including related to health settings and complex interventions. One example is the Consolidated Framework for Implementation Research. | We strengthened a theoretical framework of the paper with a section on a study design where a three-step methodological framework was suggested, including the analysis of a) objectives and design of the Program, b) its implementation, c) outcomes. The entire paper has been re-arranged according to this framework.  We discussed the possibility to use a standard framework for the implementation research but found it uncomfortable for the analysis of both design and implementation. With all advantages of this framework, it requires a totally new paper. |
| **Results section** | |
| Objectives and design section is very descriptive, but again lacks structure on the analytical level. Some major domains/themes could be identified as important factors/indicators related to implementation. | We structured this section. Major themes were identified and presented in subsections. We also strengthened the link between implementation and design sections. |
| It seems like Objective and design subsection should go under Results section as it was one objective of this study. | Done |
| It is not clear where (103) face-to-face interviews' results were reported. Some of the citations are provided in the Discussion but do not appear in the Results. | Some citations from interviews of health managers are given on page 12 of the revised paper (in Results).  Corrected a description of the survey of 103 physicians (in Methods). |
| **Discussion** |  |
| This section provides more information about the Program but does not have any discussion of the actual research performed, its strengths, weaknesses and limitations. The issues related to generalizability of the findings, selection and report biases should be discussed. | We moved some material from discussion to results section.  Added:  -the points about obstacles to the Program,  -the limitations of the research  -report biases  -generalizability of the findings  We also make a point that the limitations of the Program are deeply rooted in the weaknesses of the health system governance, particularly the lack of democratic institutions of decision-making. |
| **Overall comments** |  |
| It is an interesting paper, very relevant to the Russian health care context. It could be greatly improved if it was backed up by a theoretical framework related to the implementation research and by providing more structured and clear methodology. The paper lacks a discussion of the Limitations of the study. | We are not sure that we use a theoretical framework that you mean, but we suggest a methodological framework, which matches the objective of the paper. We got familiar with CFIR, found it useful. Plan to use it in our future research.  Thanks again for comments and recommendations. |
